# Losses of Foliage to Defoliating Insects Increase with Leaf Damage Diversity Due to the Complementarity Effect

**DOI:** 10.3390/insects16020139

**Published:** 2025-01-31

**Authors:** Mikhail V. Kozlov, Vitali Zverev

**Affiliations:** Department of Biology, University of Turku, 20014 Turku, Finland; vitzve@utu.fi

**Keywords:** complementarity effect, diversity–herbivory relationship, diversity of leaf damage, environmental gradients, insect herbivory, research methodology

## Abstract

The functioning of ecosystems critically depends on biodiversity. However, the effects of herbivore diversity on plant damage caused by herbivore feeding remain underexplored. In this study, we tested the prediction that relative losses of foliage to defoliating insects increase with leaf damage diversity (LDD), and we also explored the mechanisms underlying the observed LDD patterns. We measured insect herbivory in 501 individuals of three deciduous woody species (*Betula pubescens*, *Salix phylicifolia*, and *Vaccinium uliginosum*) across 38 localities in north-western Russia, collected 8844 leaves damaged by defoliating insects, classifying the 21,073 feeding events observed in these leaves into 29 damage types. Overall, LDD significantly decreased with increasing latitude but showed no variation along elevation or pollution gradients. Herbivory weakly but significantly increased with increasing LDD, and a strong positive correlation between the rarefied number of leaf damage types and their evenness provided evidence for the complementarity effect underlying this herbivory increase, indicating that insects producing different leaf damage types differ in their resource use.

## Simple Summary

Biodiversity sustains healthy ecosystems, but scientists have yet to fully understand how herbivore diversity affects plant damage resulting from their feeding. In this study, we tested whether plants lose more foliage when herbivores create a greater variety of feeding patterns. We analysed insect herbivory on three woody plant species across north-western Russia by examining over 8800 leaves and identifying 29 types of feeding damage. We observed that leaf damage diversity declined at higher latitudes but remained consistent across elevation and pollution gradients. Higher damage diversity corresponded to slightly increased herbivory because herbivores with diverse feeding styles complemented each other, making better use of plant resources and driving this increase in foliage loss.

## 1. Introduction

Decades ago, the scientific community reached a consensus that ecosystem functioning depends critically on biodiversity [1]. However, the biodiversity–function relationships show variability [2] because biodiversity both responds to and influences ecosystem functioning. Furthermore, the individual components of biodiversity (e.g., species richness, species composition and evenness) can have different effects on ecosystem processes [3]. The uncertainties in understanding diversity effects on these processes are particularly great when considering the diversity of consumers [1], especially herbivores [4], as this diversity is regulated by multiple feedbacks between trophic levels [5] at both ecological and evolutionary time scales.

Ecological studies exploring the relationships between biodiversity and herbivory tend to focus on the effects of plant diversity on biomass loss to insects [6,7,8], whereas the effects of herbivore diversity on the level of herbivory remain underexplored [9]. Although several studies indicate that a higher consumer species richness increases resource consumption [10], specific consumers within a community, rather than the overall consumer richness, can have a greater impact on ecosystem functioning [11]. This is especially true when a mass occurrence of a single herbivore species causes severe plant damage [12,13].

The acute shortage of information on the relationships between the diversity of insect herbivore assemblages and the total loss of plant biomass resulting from their feeding is largely related to methodological issues. Insect herbivory, which is typically quantified as a proportion of leaf area or biomass consumed by insects, can be easily measured [14,15]. In contrast, an adequate assessment of the diversity of herbivores (except for leafminers) in natural ecosystems is labour intensive, as it requires the quantitative collection of herbivores during multiple surveys in different seasons [16,17]. Furthermore, in situ observations or laboratory feeding experiments are needed to distinguish between occasional visitors and species feeding on the plants from which they were collected [18,19]. Finally, the challenges of species-level identification of all collected herbivores, especially for immature stages, have forced researchers either to use a morphospecies concept [20] or to invest time and resources in rearing collected larvae to adult stages for accurate taxonomic identification [19]. Tackling these challenges requires the development of a handy method for the rapid assessment of herbivore diversity that can be performed simultaneously with measurements of herbivory.

The common use of damage-type diversity as a proxy for herbivore diversity in paleoecology [21,22,23] is strongly supported by the significant within-tree correlation between the numbers of leaf-chewing insect species and the leaf damage types (DTs) [20]. Nevertheless, leaf damage diversity (LDD) is rarely used in studies of contemporary ecosystems (however, see [10,24,25]), and the factors affecting LDD, as well as the LDD effects on ecosystem functioning, remain insufficiently understood.

Here, we endeavoured to bridge this knowledge gap and its complication of the interpretation of palaeoecological findings by exploring the relationships between LDD imposed by defoliating insects and the total losses of leaf area attributable to these insects in contemporary ecosystems. We selected sharp environmental gradients as the focus of our study to capture the maximum possible variations in both herbivory and herbivore diversity. By doing so within a relatively compact and well-studied geographic region (north-western Russia), we ensured the study’s feasibility while maximising its ecological and environmental relevance. We first evaluated whether LDD, as quantified by the Shannon diversity index, changes with environmental conditions along elevational, latitudinal and pollution gradients. We then tested whether LDD is associated with foliar losses to insects that cause the observed damage. Finally, we asked which of the two main mechanisms—dominance of species with particular traits or complementarity among species with different traits—would best explain the observed associations between LDD and herbivory.

The first mechanism, called the ‘species identity effect’ or ‘sampling effect’, arises when a species-rich community shows an increased probability of the occurrence of an impactful species that consumes significantly more resources than an average species. The second mechanism, called the ‘species complementarity effect’, results from the exploitation of different resources by co-occurring species, so that total consumption increases with species number [11,26]. In the present study, we distinguished between these mechanisms, which are not mutually exclusive, by analysing the relationships between rarefaction-corrected numbers and the evenness of leaf DT. We proposed that a negative correlation would demonstrate the leading role of the species identity effect, whereas a positive correlation would indicate the dominance of the complementarity effect.

## 2. Materials and Methods

### 2.1. Study Plants

We explored associations between LDD and herbivory in three deciduous woody species widely distributed in northern Europe: downy birch (*Betula pubescens* Ehrh.), tea-leaf willow (*Salix phylicifolia* L.) and bog bilberry (*Vaccinium uliginosum* L.). In the Kola Peninsula, downy birch is represented by its northern variety, mountain birch (*Betula pubescens* var. *pumila* (G. Zanoni ex Murray) Govaerts). The three selected species belong to different growth forms (tree, high shrub and low shrub, respectively), differ in average leaf area (5–25, 3–12 and 0.5–2 cm^2^, respectively) and are targeted by distinct communities of herbivorous insects [27,28,29,30].

### 2.2. Study Sites

**Figure 1:**
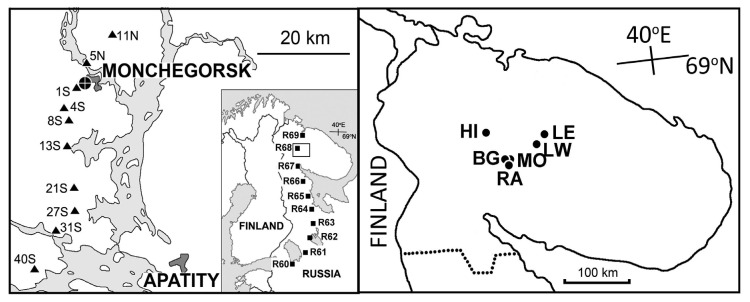
Approximate locations of study sites: triangles, pollution gradient; squares, latitudinal gradient; circles, elevation gradient. For coordinates of study sites, consult Table S1.

The elevation gradient included 18 sites (three sites in each of six groups) located from 190 to 630 m above sea level on the slopes of the Monche-tundra, Khibiny and Lovozero mountains in the central area of the Kola Peninsula, north-western Russia (Figure 1, Table S1). Six of these sites were selected in the alpine tundra at the upper distribution limit of mountain birch and tea-leaf willow. Another six were located in subalpine birch woodlands, while the remaining six were located in closed-canopy coniferous forests. Elevation (m) above sea level was used as a proxy of environmental stress in this gradient. For additional details, including photographs of several sites, see [31].

The pollution gradient consisted of 10 sites located 1–40 km from the Monchegorsk nickel–copper smelter in the Kola Peninsula and ranged from extremely polluted industrial barrens to near-pristine Norway spruce forests (Figure 1, Table S1). One of these sites (coded 4S) is identical to the HIF site from the elevational gradient. The concentration of nickel (the main metal pollutant emitted by the Monchegorsk smelter; μg g^−1^) in birch foliage was used as a proxy of environmental stress in this gradient. For additional details, including the history of pollution impact and photographs of several sites, see [32,33].

The latitudinal gradient comprised 10 sites between 60° N near St. Petersburg and 69° N near Murmansk (Figure 1, Table S1). One of these sites (coded R68) is identical to the site 11N from the pollution gradient. Latitude (° N) was used as a proxy of environmental stress in this gradient. All sites were located in unevenly aged, unmanaged old-growth forests dominated by Scots pine (*Pinus sylvestris* L.), Norway spruce (*Picea abies* (L.) Karst.) and downy birch. The field layer vegetation was dominated by bilberry (*Vaccinium myrtillus* L.), with significant contributions from crowberry (*Empetrum nigrum* L.) and lingonberry (*V. vitis-idaea* L.). For additional details, see [34].

### 2.3. Sampling

The branches used for the measurement of herbivory and the assessment of LDD were collected in the early autumn of 2014 (elevation gradient: 12–15 August; pollution gradient: 12–17 August; latitudinal gradient: 17–22 August) after most insect herbivores had completed their feeding. We aimed to sample one branch containing 100–200 leaves from each of five mature individuals of each study species at each site, haphazardly selecting individuals that were spaced at least 10 m apart from others of the same species. However, some species were infrequent or even completely missed in some locations, which affected the sample sizes. To minimise unconscious selection bias, we maintained a distance of 5–10 m from the plants during branch selection.

### 2.4. Measurement of Herbivory

In the laboratory, the first 100 leaves (starting from the tip of the branch) were categorised into the following damage classes based on a visual assessment of the percentage of the leaf area that was consumed or damaged (e.g., skeletonised) by defoliating insects: 0% (intact leaves), 0.01–1%, 1–5%, 5–25%, 25–50%, 50–75% and 75–100%. To calculate the herbivory level, we multiplied the number of leaves in each damage class by the median percentage of the respective class (0.5% for 0.01–1%, 3% for 1–5%, etc.). The resulting values were summed across all damage classes and divided by the total number of leaves in the sample [14,35].

### 2.5. Classification of Damage Types

We restricted our study to defoliating insects, including both species that feed externally on foliage and those that feed within shelters (e.g., leafrollers). We developed an original classification of DTs because many DTs observed by us were missing in the classification developed for fossil records [23,36]. Following the approach used in previous studies [10,20,25,36], we subdivided DTs produced by defoliating insects into four feeding types: skeletonisation (SK), margin feeding (MF), hole feeding (HF) and shelter feeding (SF). Each of the 29 DTs recognised in this study are defined by a diagnostic suite of characters outlined in Text S1 and illustrated by photographs of damaged leaves and by drawings of shelters made from leaves (Figures S1–S3). Defining attributes of DTs within each feeding type included size, shape and position on the leaf. Only two of the considered DTs were unambiguously associated with a single insect species: SF1 is produced by females of the beetle *Deporaus betulae* (L.) and SK8 results from the feeding of larvae of a moth *Bucculatrix demaryella* (Dup.) after they leave their mines.

### 2.6. Assessment of Leaf Damage Diversity

Leaves with feeding marks from defoliators (1–44 from each branch, depending on the availability of leaves damaged by the targeted insects: median number 24) were used to assess LDD. Each individual damage mark (feeding event, hereafter) on each leaf was attributed to one of 29 DTs by the same person (M.V.K.), and the numbers of each DT were recorded for each leaf (Data S1).

### 2.7. Data Analysis

Tree-specific values were calculated by summing feeding events across all collected leaves (Data S2). Data from trees with 1–4 feeding events were disregarded. LDD was quantified using the Shannon diversity index based on the number of feeding events for each DT. The values of this index were compared among plant species using ANOVA (SAS GLM procedure; [37]), whereas occurrences of feeding types were compared using the χ^2^ test (SAS FREQ procedure). The evenness was measured using the Pielou’s index. The rarefied numbers of DTs were calculated for samples of 25 feeding events for site-specific data and 1000 feeding events for species-specific data using the PAST programme [38]. Non-linear patterns in the data were searched for using quadratic regression analyses (SAS REG procedure). Herbivory values were log (x + 0.1)-transformed prior the analyses. The Pearson linear correlation coefficients were separately calculated for each species-by-gradient combination based on the site-specific means (SAS CORR procedure) and then *z*-transformed and weighted by sample sizes to obtain the *z*_r_ effect size (ES) values. The mean ESs were computed and compared using random-effects models in the MetaWin 2.0 programme [39]. An effect was deemed to be statistically significant if the bootstrap 95% confidence interval (CI95) of the mean ES did not include zero. Variations in the ESs among environmental gradients and among plant species were analysed using the heterogeneity index *Q*_B_, tested against the χ^2^ distribution.

## 3. Results

### 3.1. Classification and Occurrences of Leaf Damage Types

We analysed 8844 leaves containing 21,073 feeding events. We classified this damage (Data S1) into 29 DTs belonging to four feeding types: skeletonisation (8 DTs, 7453 events), margin feeding (10 DTs, 12,399 events), hole feeding (3 DTs, 1033 events) and shelter feeding (8 DTs, 188 events) (Text S1, Figures S1–S3). The maximum leaf-specific values were 33 feeding events and six DTs (Data S1).

### 3.2. Differences Among Plant Species in Leaf Damage Types

Among the 29 DTs, 21 were shared across the studied plant species. Two DTs (SK8 and SF1) were observed only in *B. pubescens*, one (SK6) only in *S. phylicifolia* and one (MF6) only in *V. uliginosum*. A random sample of 1000 leaves of *B. pubescens* contained (mean ± S.E.) 22.75 ± 1.33 DTs, of *S. phylicifolia* 20.77 ± 1.15 DTs, and of *V. uliginosum* 20.59 ± 1.07 DTs. Although among-species differences in the rarefied number of DTs were not statistically significant (*p* > 0.20), the differences in Shannon diversity index (*B. pubescens*: 1.36 ± 0.05, *n* = 38 sites; *S. phylicifolia*: 1.50 ± 0.05, *n* = 28 sites; *V. uliginosum*: 1.33 ± 0.04, *n* = 37 sites) were significant (*F*_2,100_ = 3.42, *p* = 0.04).

The plant species differed significantly (*χ*^2^ = 594.5, df = 6, *p* < 0.0001) in regard to feeding type occurrences. The sum of feeding events attributed to skeletonisation and margin feeding ranged from 92–97% across all plant species, but their ratio was much greater in *B. pubescens* (65% to 30%) than in *S. phylicifolia* (51% to 41%) and *V. uliginosum* (56% to 41%). The two latter species also differed in their ratios of hole feeding to shelter feeding, which varied from 1.49% to 1.40% in *V. uliginosum* and from 8.13% to 0.25% in *S. phylicifolia*.

### 3.3. Leaf Damage Diversity Along Environmental Gradients

**Figure 2:**
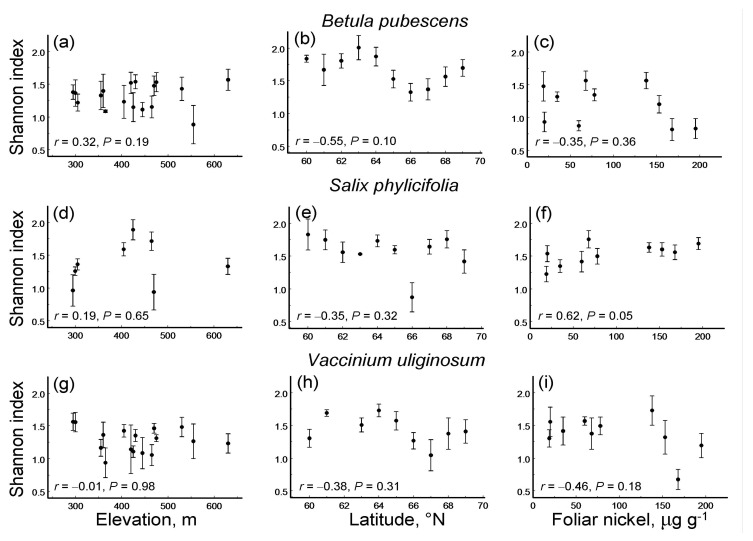
Changes in the diversity of leaf damage (Shannon index, mean ± S.E.; dots = means, bars = standard errors; median *n* = 5 individuals for each site by plant species combination) in three plant species along three environmental gradients (Pearson linear correlation coefficients and probability levels): (**a**–**c**) *Betula pubescens*; (**d**–**f**) *Salix phylicifolia*; (**g**–**i**) *Vaccinium uliginosum*; (**a**,**d**,**g**) elevation gradient; (**b**,**e**,**h**) latitudinal gradient; (**c**,**f**,**i**) pollution gradient. Proxies of environmental stress in these gradients: elevation (m) above sea level, latitude (° N) and the concentration of nickel (μg g^−1^) in birch foliage, respectively.

Only one of the nine species-by-gradient combinations showed a significant linear pattern in LDD: in *S. phylicifolia*, the Shannon diversity index based on DTs increased with the pollution level (Figure 2). None of these nine combinations showed a significant quadratic pattern (*p* = 0.13 … 0.97).

**Figure 3:**
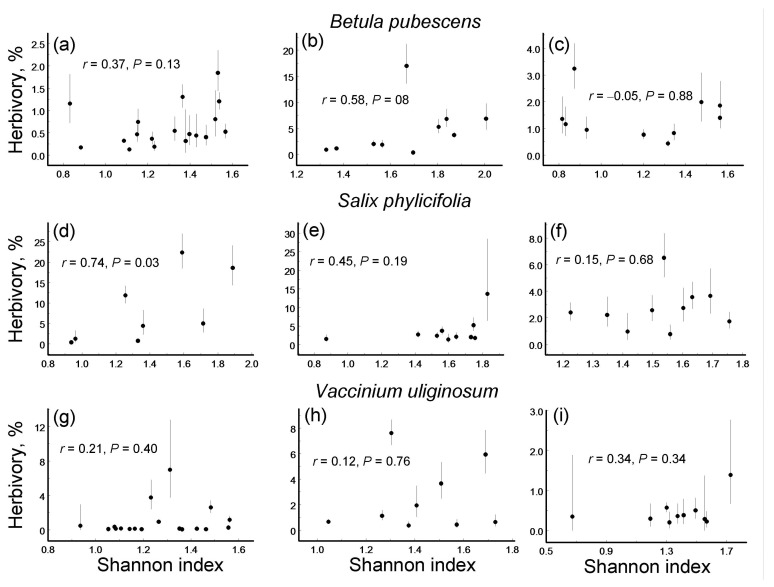
Correlations (Pearson linear coefficients based on log (x + 0.1)-transformed values and probability levels) between herbivory (back-transformed mean ± S.E.; dots = means, bars = standard errors; median *n* = 5 individuals for each site by plant species combination) and diversity of leaf damage (Shannon index) in three plant species within three environmental gradients: (**a**–**c**) *Betula pubescens*; (**d**–**f**) *Salix phylicifolia*; (**g**–**i**) *Vaccinium uliginosum*; (**a**,**d**,**g**) elevation gradient; (**b**,**e**,**h**) latitudinal gradient; (**c**,**f**,**i**) pollution gradient.

The ES based on correlation coefficients between LDD and the values of environmental stress proxies did not differ significantly among either plant species (*Q*_B_ = 1.31, df = 2, *p* = 0.54) or environmental gradients (Figure 5a). Overall, LDD did not change with the level of environmental stress. Nevertheless, across all three plant species, LDD significantly decreased with an increase in latitude, did not change with pollution and tended to increase with an increase in elevation (Figure 5a). However, the rarefaction-corrected number of DTs decreased only by 7.6% from 60° N to 69° N, and this decrease was far from statistically significant (ES = −0.18; *n* = 3 species; CI95 = −0.35 … 0.17).

### 3.4. Leaf Damage Diversity and Herbivory

Only one of the nine species-by-gradient combinations showed a significant correlation between herbivory and LDD: in *S. phylicifolia*, this correlation was significantly positive for the elevation gradient (Figure 3).

The ES based on correlation coefficients between herbivory and LDD did not differ significantly either among plant species (*Q*_B_ = 0.71, df = 2, *p* = 0.54) or among environmental gradients (Figure 5b). Overall, herbivory increased with an increase in LDD, although this association was statistically significant only in the latitudinal and elevational gradients (Figure 5b).

**Figure 4:**
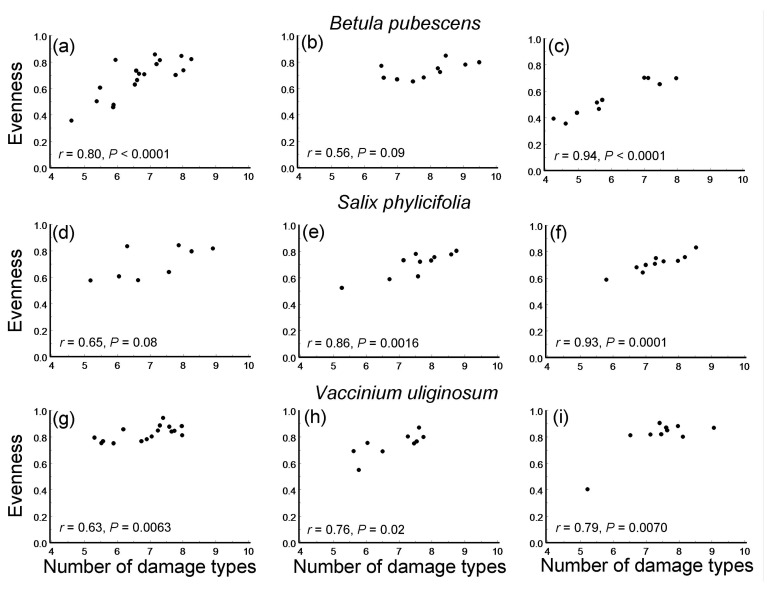
Correlations (Pearson linear coefficients and probability levels) between the evenness of damage types and their rarefaction-corrected numbers in three plant species within three environmental gradients: (**a**–**c**) *Betula pubescens*; (**d**–**f**) *Salix phylicifolia*; (**g**–**i**) *Vaccinium uliginosum*; (**a**,**d**,**g**) elevation gradient; (**b**,**e**,**h**) latitudinal gradient; (**c**,**f**,**i**) pollution gradient.

### 3.5. Number and Evenness of Leaf Damage Types

**Figure 5:**
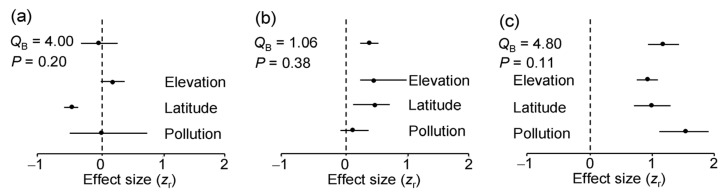
Meta-analysis of effect sizes based on correlation coefficients: (**a**) between the diversity of leaf damage and a proxy of environmental stress; (**b**) between log (x + 0.1)-transformed herbivory and the diversity of leaf damage; (**c**) between evenness of damage types and their rarefaction-corrected numbers. The mean values (dots) are each based on 9 (overall effect) or 3 (gradient-specific) effect sizes; horizontal lines denote bootstrap 95% confidence intervals (CI95). An effect is statistically significant if CI95 does not include zero (indicated by dashed vertical line).

The evenness of the DTs significantly increased with an increase in the rarefaction-corrected number of DTs in seven of the nine species-by-gradient combinations (Figure 4). Consequently, both the overall ES and all the gradient-specific ESs based on correlations between these variables were highly statistically significant (Figure 5c) and showed no variation among either plant species (*Q*_B_ = 1.50, df = 2, *p* = 0.50) or environmental gradients (Figure 5c).

## 4. Discussion

### 4.1. Numbers of Leaf Damage Types and Insect Herbivore Species

We identified 29 leaf DTs across the three plant species (*B. pubescens*, *S. phylicifolia* and *V. uliginosum*) growing in diverse habitats spanning 10 degrees of latitude and 630 m in elevation. This number approaches the 41 DTs identified in fossil leaves for hole feeders, margin feeders and skeletonisers and exceeds the typical dozen or two DTs reported for fossil floras examined for insect herbivory [40]. This result likely reflects our large sample size (8844 damaged leaves) and the inclusion of eight DTs caused by shelter-feeding insects.

Good knowledge of the insect fauna of Finland, which neighbours our study region and encompasses a similar range of latitudes and elevations, enables comparisons betrween DT numbers and the species richness of herbivorous insects. The larvae of at least 158 moth and butterfly (Lepidoptera) species feed externally on the leaves of our study plants in Finland [30]. By combining host plant data [29] with distribution data (obtained from laji.fi), we identified 37 leaf beetle (Chrysomelidae) species feeding on the same plants in Finland. Data for sawflies (Tenthredinidae), another major group of external leaf feeders, suggest that 80–90 species feed on study plants in Finland (M. Mutanen, pers. comm.). Thus, the total number of insect species feeding on *B. pubescens*, *S. phylicifolia* and *V. uliginosum* in the study region likely exceeds the observed number of leaf DTs by at least an order of magnitude.

Notably, only 8 of the 158 Lepidoptera species (5.1%), 2 of the 37 Chrysomelidae species (5.4%), and 1 of the 80–90 Tenthredinidae species (1.2%) fed on all three study plants. These numbers clearly contrasted with the percentage of shared DTs (72.4%), confirming that multiple insect species, potentially from diverse taxonomic groups, produce similar DTs—a phenomenon documented in both contemporary and fossil ecosystems [20,23].

Our findings stress the need to create a more elaborate and detailed classification system for DTs, possibly involving the quantitative characteristics of feeding damage to decrease DT redundancy. This task requires the archiving of the images of all leaves with traces of herbivory that will be studied in the future in digital repositories and the development of an objective and automated method that incorporates AI-driven object recognition for identifying DTs.

### 4.2. Shelter Feeders: A Promising Group for Studying Leaf Damage Diversity

Certain insects, particularly the larvae of moths and butterflies, construct conspicuous shelters at their feeding sites [41]. However, these shelters, such as leafrolls, are relatively uncommon in the fossil records due to detection challenges [42], potentially because the shelters of leaf-tying herbivores decompose faster than untied leaves damaged by free-living insects [43].

Although shelter feeders are not typically considered in paleoecology [23], this type of damage appears promising for examining LDD in contemporary ecosystems. Shelters exhibit a wide range of shapes and structures, from simple folded leaf fragments to intricate silk tunnels and multi-leaf constructions. Leaves may be rolled, folded or tied to each other or to twigs or fruits [44,45,46,47]. Although shelter feeders were responsible for only 0.88% of the feeding events in our samples, they were represented by eight distinct DTs. This suggests that DT redundancy may be lower among shelter-feeding insects than among skeletonisers, hole feeders or margin feeders. Consequently, we recommend paying particular attention to shelter feeders when using LDD to study the diversity of herbivorous insect communities in present-day ecosystems.

### 4.3. Leaf Damage Diversity and Herbivore Diversity Along Environmental Gradients

The changes in LDD observed along environmental gradients align broadly with previously documented patterns in taxonomic (alpha) diversity. The pronounced decrease in LDD with increasing latitude corresponds to the poleward decline in biodiversity noted both in the same latitudinal gradient [34] and at global scales [48,49]. However, the rarefaction-corrected number of DTs, commonly used in paleoecology as a proxy for species richness [23], showed no significant latitudinal trend. This result contrasts with the pronounced decline observed in herbivore species richness between southernmost Finland (provenances Ab, N and K, matching our sites R60 and R61 by latitudes) and northernmost Finland (provenances Le and Li, matching our sites R68 and R69 by latitudes). Distribution data (obtained from laji.fi) revealed that the number of moth and butterfly species declined between these regions from 155 to 122, while the number of leaf beetle species feeding on our study plants decreased from 36 to 25. These reductions, amounting to declines of 21.3% and 30.6% species, respectively, are three to four times greater than the observed 7.3% decline in the average number of DTs. This discrepancy likely reflects the DT redundancy discussed earlier and underscores the importance of selecting appropriate diversity metrics. In this context, the Shannon index seems to bve a more effective measure of LDD than the number of DTs.

The lack of an effect of industrial pollution on LDD in our study is consistent with the results from meta-analysis [50] even though the diversity of moths and butterflies—a taxon dominated by herbivores—decreased significantly along our pollution gradient [51]. The general pattern of elevational effects on diversity remains unclear, as both the shape and direction of elevation–diversity relationships vary across taxa [52]. Our finding of no significant correlation between elevation and LDD falls within this range of reported variation. Taken together, our observations suggest that, despite multiple limitations, LDD could serve as a coarse proxy for the taxonomic diversity of insect herbivores in ecological and environmental studies of present-day ecosystems.

### 4.4. Leaf Damage Diversity and Herbivory: Patterns and Mechanisms

For decades, studies on the consumer diversity–resource consumption relationship have focused on predator–prey dynamics or aquatic systems [53]. In contrast, the presence of similar patterns in terrestrial plant–herbivore systems remains largely unexplored [11]. This gap justifies the inclusion of consumers as a critical frontier in studies linking biodiversity to ecosystem functions [4].

Previous research has reported varying relationships between herbivore species richness and plant losses to herbivory. For example, habitat fragmentation reduced herbivore species richness but did not affect herbivory levels [54]. Similarly, while herbivore species richness changed with latitude from Mexico to Bolivia, herbivory levels did not [55]. In contrast, urbanisation was shown to reduce both LDD and herbivory [25].

Our study is the first to demonstrate that herbivory levels in natural ecosystems are positively associated with LDD, as quantified by the Shannon diversity index. This index captures both of the key components of LDD: the number of DTs (analogous to species richness) and the evenness of the feeding event distribution among DTs. Although our conclusions are based on herbivory–LDD relationships within three individual plant species, the consistency of the patterns across these species, which span different plant families and growth forms, and the lack of differences between natural (elevational, latitudinal) and human-induced (pollution) gradients, supports the generalisability of our findings to a broader context.

Our finding that increasing herbivory with higher LDD is driven by species complementarity rather than species identity aligns with the experiment that discovered complementarity among grassland herbivores in terms of their impact on plant biomass [56]. At the same time, our finding contrasts with the dominant role of identity effects reported in prior studies [53]. Further research is necessary to determine whether this disagreement in mechanisms between study systems arises from fundamental differences between predators and herbivores or between aquatic and terrestrial ecosystems.

## 5. Conclusions

We utilised the diversity of leaf damage types (LDD), a measure often overlooked in contemporary herbivory studies, to enhance our understanding of the consumer diversity–resource consumption relationship in terrestrial ecosystems. Our findings revealed that insect herbivory increased weakly, but significantly, with higher LDD. Notably, the strong positive correlation between the rarefaction-corrected number of leaf damage types and their evenness supported the complementarity effect, indicating that insects producing different leaf damage types differ in their resource usage. Despite the current limited understanding of the factors influencing the composition, quantity and relative abundances of plant damage types in present-day ecosystems, we conclude that LDD is a valuable, albeit coarse, proxy for herbivore diversity in contemporary ecological research.

## Data Availability

All data are included in this publication as Supplementary Materials.

## Supplementary Materials

The following supporting information can be downloaded at: https://www.mdpi.com/article/10.3390/insects16020139/s1, Table S1: Characteristics of study sites. Text S1: Description of leaf damage types. Figure S1: Leaf damage types: skeletonisation (SK1–SK8). Figure S2: Leaf damage types: marginal feeding (MF1–MF8). Figure S3: Leaf damage types: marginal feeding (MF9–MF10), hole feeding (HF1–HF3) and shelter feeding (SF1–SF8). Data S1: Occurrence of damage types in individual leaves. Data S2: Tree- and plot-specific data.
